# My general manager is warmer than department manager: Stereotypes about senior and junior high-power individuals and their influences on spontaneous trait inference

**DOI:** 10.3389/fpsyg.2022.1015736

**Published:** 2022-12-01

**Authors:** Feng Yang, Minyan Li, Yang Han, Xinru Fan, Qing Zhang

**Affiliations:** ^1^Teacher Education Department, Taishan University, Tai’an, Shandong, China; ^2^The Department of Psychology, Beijing Sport University, Beijing, China; ^3^The Department of Politics and Public Administration, Shandong Youth University of Political Science, Jinan, Shandong, China

**Keywords:** power, stereotype, spontaneous trait inference, stereotype content model, social cognition, impression formation

## Abstract

**Introduction:**

Previous research suggests that high-power (HP) individuals are stereotyped as positive competence but negative warmth.

**Object:**

By subdividing HP individuals into junior and senior HP individuals, the current research conducted five studies to examine the warmth perception differences toward senior and junior HP individuals in Confucian culture and the downstream effects on spontaneous trait inference (STI).

**Method and results:**

By using different paradigms, Study 1 and 2 found that participants tended to perceive junior HP individuals as negative on the warmth dimension and perceive senior HP individuals as positive on the warmth dimension. The following Study 3 and 4 further found that the warmth perception difference toward senior and junior HP individuals had an influence on STI. Specifically, participants were inclined to make STI from behaviors implying negative warmth traits when behavioral actors were junior HP individuals while they were inclined to make STI from behaviors implying positive warmth traits when behavioral actors were senior HP individuals. Additionally, Study 4 found that perceived social responsibility about HP individuals accounted for the power stereotype effects in STI, the more social responsibility participants perceived about senior HP individuals, the stronger power stereotype effects they showed in STI. The final Study 5 revealed that the different power stereotype effects in STI induced by senior and junior HP actors were observed only in Confucian culture, but not in non-Confucian culture.

**Conclusion:**

The present research firstly demonstrated that the warmth perceptions about senior and junior produced different influences on STI in Confucian culture, and also enriched the understanding about the culture-specificity of the stereotype content model.

## Introduction

When people read the sentence “the secretary solved the mystery halfway through the book,” they may spontaneously infer the trait “clever” (Winter and Uleman, 1984). Researchers term this phenomenon as spontaneous trait inference (STI), which refers to the trait inferences occurring in the absence of explicit intention to infer traits or form an impression of that person (Uleman et al., 1996). Despite that automaticity is a salient feature of STI, previous research still reveals a variety of factors affecting the occurrence of STI, including behavioral actors’ power (Rim et al., 2009; Uleman et al., 2012; Wang and Yang, 2017). Not long ago, Wang and Yang (2017) found that the power label of behavioral actors could activate the corresponding power stereotypes, high-power (HP) individuals were stereotyped as positive on the competence dimension but negative on the warmth dimension and low-power (LP) individuals were stereotyped as negative on the competence dimension but positive on the warmth dimension. As a result, participants were more likely to make STI from behaviors implying negative warmth traits than behaviors implying positive warmth traits when behavioral actors were HP individuals (Wang and Yang, 2017).

Notably, recent research by Li et al. (2022) indicates that dividing power into HP and LP in a dualistic way may oversimplify the representation about power in real life, especially for the representation about HP individuals. Their research demonstrates that in Confucian culture, when HP individuals are subdivided into senior and junior HP individuals, people actually possess different warmth perceptions for them—on the relative level, senior HP individuals were perceived as positive on the warmth dimension and junior HP individuals were perceived as negative on the warmth dimension. According to the definition by Li et al. (2022), junior HP individuals refer to those HP individuals who are primarily responsible for directly managing ordinary people in a society or employees in a company (e.g., department manager), and senior HP individuals refer to those HP individuals who are primarily responsible for managing junior HP individuals (e.g., general manager).

It should be pointed out, although Wang and Yang (2017) have examined the effects of power stereotypes on STI and found that participants were more likely to make STI from behaviors implying negative warmth traits than behaviors implying positive warmth traits when behavioral actors were HP individuals, the difference about the warmth perception of senior and junior HP individuals revealed by Li et al. (2022) raise the question whether power stereotypes about senior and junior HP individuals will produce different effects on STI in Confucian culture. The answer to this question not only contributes to more comprehensively and accurately assessing the relationship between power stereotypes and STI, but also helps us firstly explore whether the subtle difference between senior and junior HP stereotypes will have an influence on individuals’ implicit impression formation about others. Given that, the current research would examine whether there were any differences about the warmth perception toward senior and junior HP individuals, and how such different warmth perceptions affected the occurrence of STI.

## Literature review

### The flexibility of STI

For a long time, the automaticity of STI has received great attention from researchers (Rim et al., 2009; Uleman et al., 2012; Shimizu et al., 2017). According to the four criteria of automaticity proposed by Bargh (1994)—lack of awareness, lack of intention, cognitive efficiency, and lack of control, the occurrence of STI obviously can be defined as an automatic process. It can occur when participants were asked to remember trait-implying sentences or even just read such sentence (Uleman et al., 1996). Moreover, in most cases, participants actually did not realize the occurrence of such trait inferences (Winter and Uleman, 1984). In addition, prior literature suggests that the occurrence of STI requires little cognitive capacity and participants still could make STI when participants were assigned under high cognitive load (Todorov and Uleman, 2003). Despite the highly automatic characteristics of STI, a great deal of research still demonstrates that the occurrence of STI is a flexible process and a variety of factors relevant to both perceivers and actors can affect the occurrence of STI (for a review, see Uleman et al., 2012; also see Rim et al., 2009).

Wigboldus et al. (2003) has pointed out that in real life, people not only put focus on processing behaviors, but also pay close attention to some salient features of actors, such as skin color, gender, age and so on. Supporting the proposition by Wigboldus et al. (2003), a large body of empirical research suggests that when people draw STI from trait-implying behaviors, some salient social labels of actors can activate the corresponding stereotypes, thus affecting the occurrence of STI (Wigboldus et al., 2004; Ramos et al., 2012; Wang and Yang, 2017). For example, as an important social label, the gender of actors can automatically activate gender stereotypes of perceivers within a very short time (Banaji and Hardin, 1996). And the activation of stereotypes in turn enhances the accessibility of stereotype-consistent traits and inhibits the accessibility of stereotype-inconsistent traits (Dijksterhuis and van Knippenberg, 1996). As a consequence, compared to gender stereotype-inconsistent behaviors, perceivers are more likely to make STI from gender stereotype-consistent behaviors (Yan et al., 2012). So far, researchers have revealed multiple kinds of stereotype effects in STI, including race, age, gender, and power stereotypes (Wigboldus et al., 2003, 2004; Ramos et al., 2012; Yan et al., 2012; Wang et al., 2015; Wang and Yang, 2017).

### Power stereotypes and confucianism

Power is a basic aspect of social life and has profound effects on our psychological and behavioral processes (Fiske and Dépret, 1996; Magee and Galinsky, 2008). In social psychology field, researchers generally define power as relative capacity to modify others’ states by providing/withholding resources or administering punishments (Keltner et al., 2003). Power in essence means asymmetric control or dependence—LP individuals depend on HP individuals but HP individuals depend less on LP individuals (Keltner et al., 2003; Lammers et al., 2012). To get valuable resources for survival, LP individuals remain high affiliation motivation toward others surrounding them, especially toward HP individuals (Magee and Smith, 2013; Magee, 2020). So, LP individuals in theory should display positive warmth trait in interpersonal communications so that they can get necessary resources. In contrast to LP individuals, HP individuals almost can satisfy their various needs without the assistance of LP individuals, so they are less motivated to affiliate with others (Inesi et al., 2012; Overbeck and Droutman, 2013). Correspondingly, they in theory should display relatively negative warmth traits in communication with others. Consistent with the above reasoning based on the asymmetrical hypothesis, through the extensive measurements of various groups in society, the Stereotype Content Model (SCM) proposed by Fiske et al. (2002) also reveals that the members belonging to HP group are considered to display negative warmth traits and the members belonging to LP group are considered to display positive warmth traits (Fiske et al., 2002, 2007; Cuddy et al., 2008).

It should be pointed out that, although an extensive body of research across the world demonstrates that HP individuals are perceived as negative warmth, recent research by Li et al. (2022) suggests that, in Confucian culture, there still may be some variations about the warmth perception of HP individuals when HP individuals are further classified into senior and junior HP individuals. In the research, researchers employed the trait-rating task to detect the stereotypes about senior and junior HP individuals (Study 1). The results showed that participants indicated more positive warmth evaluations for senior HP individuals than for junior HP individuals. In the following study (Study 3), Li et al. (2022) found that when the IAT was applied to detect the implicit stereotypes about senior and junior HP individuals, participants still tended to associate senior HP individuals with positive warmth traits and associate junior HP individuals with negative warmth traits.

According to Li et al.’s (2022) explanation, the above results rooted in the power construction of Confucianism. Specifically, Confucianism has been the dominant philosophy in China since the Han dynasty, Confucianism thus produces a profound influence on Chinese people’s cognition and behaviors (Fung, 1948/2018). About how one person should exert his/her power, Confucianism shows its “double-standard” to some extent. On one hand, Confucianism contends that more power implies greater social responsibility and power should be used to serve for the whole society (Chen and Chung, 1994; Hwang, 2012). Under this logic, HP individuals in Confucian culture are expected to display positive warmth traits in communication with others. For example, Confucianism contends that an excellent governor should love ordinary people as if they were his/her own children. On the other hand, Confucianism puts an emphasis on the stateliness, status, and privilege possessed by HP individuals, and LP individuals need to pay close attention to their words and behaviors in communications with HP individuals (Chen and Chung, 1994; Chen et al., 2013). As a result, the “double-standard” of Confucian power construction may lead to negative warmth perception for the junior HP individuals with whom most people can frequently interact, whereas they tend to expect senior HP individuals—a group that they actually have less chance to contact with—to display positive warmth traits in social interactions.

We noticed that, so far, Li et al.’ (2022) research was the first and only time to demonstrate different warmth perceptions about senior and junior HP individuals. Given that, prior to examining the effects of power stereotypes on STI, we firstly examined participants’ warmth perception about senior and junior HP individuals so that we could conceptually replicate the results of previous research. We hypothesized that,

*In Confucian culture, on the relative level, senior HP individuals would be perceived as more positive on the warmth dimension in comparison to junior HP individuals* (Hypothesis 1).

### Power stereotypes, STI, and confucianism

Up to now, a large body of research has investigated the contents of power stereotypes and a series of downstream effects on individuals’ cognition and behaviors (Russell and Fiske, 2008; Fragale et al., 2009; Zhang et al., 2013; Wang and Yang, 2017). Recently, Wang and Yang (2017) classified power into HP and LP, and utilized the probe recognition paradigm to investigate the effects of power stereotypes on STI in Confucian culture. In the research, Wang and Yang (2017) manipulated the power of actors by assigning “*the leader*” or “*the subordinate*” as the subject of behavioral sentences. The results showed that when actors of behavioral sentences were denoted as “the leader,” participants were more likely to draw STI from behavioral sentences implying negative warmth traits than from implying positive warmth traits, suggesting that participants had more negative warmth perceptions for HP individuals than for LP individuals.

Notably, as we have pointed out in the beginning, classifying power into HP and LP in a dualistic way may oversimplify the influences of power stereotypes on STI (Anicich et al., 2020). Recent research has found that, in Confucian culture, when HP individuals were subdivided into senior and junior HP individuals, participants tended to have negative warmth perceptions for junior HP individuals, but tended to have positive warmth perceptions for senior HP individuals (Li et al., 2022). That means, when examining the effects of power stereotypes on STI, if HP individuals were subdivided into senior and junior HP individuals, they may produce effects on STI in opposite directions. Given that, the second goal of the current research was to extend Wang and Yang’s (2017) work by subdividing HP into senior and junior HP and investigate the effects of power stereotypes on STI in a more focused way. We hypothesized that,

*In Confucian culture, when behavioral actors were senior HP individuals, participants would be more likely to make STI from behaviors implying positive warmth traits than from behaviors implying negative warmth traits; By contrast, when behavioral actors were junior HP individuals, participants would be more likely to draw STI from behaviors implying negative warmth traits than from implying positive warmth traits* (Hypothesis 2).

In line with Li et al.’ (2022) research, we explained the expected power stereotype effects in STI in terms of Confucian power construction. As we have documented, Confucianism has been dominant philosophy in China, which alleges that more power represents more social responsibility (Fung, 1948/2018; Hill, 2006). Due to positive expectation about the social responsibility of senior HP individuals, individuals in Confucian culture may be more likely to make STI from behaviors implying positive warmth traits when behavioral actors are senior HP individuals. Relative to senior HP individuals, junior HP individuals in Confucian culture are more likely to be associated with negative warmth traits (Li et al., 2022). As a consequence, individuals in Confucian culture may also be inclined to make STI from behaviors implying negative warmth traits when behavioral actors are junior HP individuals.

Actually, this Confucianism explanation has received partial supports in Li et al.’s (2022) research which found that participants in Confucian culture (Chinese participants) expected more social responsibility for senior HP individuals than for junior HP individuals while participants in non-Confucian culture (American participants) expected equally low social responsibility for both senior and junior HP individuals. In addition, the well accepted collectivism/individualism theory also provides additional supports for the Confucianism explanation (Singelis et al., 1995; Triandis, 1995; Triandis et al., 1998; Triandis and Gelfand, 1998). Specifically, by making the vertical/horizontal dimension intersect with the traditional individualism/collectivism dimension, Triandis et al. thus have delineated four distinct cultural orientations: vertical individualism (VI), horizontal individualism (HI), vertical collectivism (VC), and horizontal collectivism (HC). By applying this classification criterion, China-Mainland should belong to VC society. However, it should be pointed out, although there are some subtle differences between HC and VC, individuals in both types of collectivistic cultures tend to emphasize common goals with others, interdependence and responsibility for others, and social sociability (Torelli and Shavitt, 2010). By contrast, individuals in both types of individualistic cultures tend to emphasize personal value and distinguish themselves from others despite that there also are some subtle differences between them. As a result, compared to HP individuals in individualistic culture, HP individuals in collectivistic society in general are expected to respond to the needs and interests of others to a greater extent (Brewer and Chen, 2007; Torelli and Shavitt, 2010). For example, in the negotiation task, participants in collectivistic society anticipated more allocations from the dominant counterparts than participants in individualistic society (Buchan et al., 2004), and due to this positive anticipation, participants in collectivistic society were also more likely to reject unfair allocations in comparison to participants in individualistic society (Li et al., 2016).

Taken together, we proposed that in Confucian culture, perceived social responsibility for senior and junior HP individuals may account for the power stereotype effects in STI. Specifically,

*In Confucian culture, the more social responsibility participants perceived about senior HP individuals (compared to junior HP individuals), the stronger power stereotype effects they showed in STI* (Hypothesis 3).

Based on Hypothesis 3, we further reasoned that if perceived social responsibility about senior HP individuals accounted for the power stereotype effects in STI under Confucian culture, the effects of power stereotypes on STI may be culture-specific. Namely, the power stereotype effects mentioned above may be observed only in Confucian culture; as for non-Confucian culture, given that Li et al. (2022) have found that American participants perceived both senior and junior HP individuals as negative on the warmth dimension, we thus speculated that individuals in non-Confucian culture may be prone to make STI from trait-implying behaviors implying negative warmth traits when behavioral actors were HP individuals regardless of senior or junior HP individuals. Overall, our Hypothesis 4 was,

*Participants in Confucian culture made stronger STI from behaviors implying positive warmth traits when actors were senior HP individuals, and made more stronger STI from behaviors implying negative warmth traits when actors were junior HP individuals, whereas participants in non-Confucian culture made stronger STI from behaviors implying negative warmth traits in both cases* (Hypothesis 4).

## The current research

On the basis of previous research (Wang and Yang, 2017; Li et al., 2022), the current research subdivided HP individuals into senior and junior HP individuals, and then examined whether and how perceived warmth differences about senior and junior HP individuals affected the occurrence of STI. To this end, we conducted five studies in the current research. In Study 1 and 2, we used the trait-rating task (Study 1) and the implicit association task (IAT, Study 2) to examine whether there were any differences about the warmth perception toward senior and junior HP individuals. In the following Study 3 and 4, we used the probe recognition paradigm and the modified false recognition paradigm to further examine the effects of senior and junior HP stereotypes on STI, and in Study 4, we also examined whether perceived social responsibility about HP individuals could account for the observed power stereotype effects in STI. In our final Study 5, we conducted an online cross-cultural study to compare whether there were any differences about the power stereotype effects in STI between Confucian and non-Confucian cultures.

## Study 1

In Study 1, we used the trait-rating task to examine whether participants would give different warmth evaluations for senior and junior HP individuals, so that we could provide initial evidence for our Hypothesis 1. The trait-rating task included two evaluated targets—*junior leader* and *senior leader*. In Chinese culture, the “junior leader” commonly refers to those junior HP individuals in general terms and the “senior leader” commonly refers to those senior HP individuals in general terms. For each of the two targets, participants needed to indicate to what extent each of the given traits was in accordance with the target. More detailed introductions about the task were provided on the *materials and procedure* section.

### Participants and design

Study 1 was a single-factor within-subjects design with two levels (senior HP vs. junior HP). According to the calculation of the G*Power 3.1 (Faul et al., 2009; a presupposed medium effect size 0.5 and being significant at the 0.05 level), we needed at least 45 participants in Study 1. Considering possible invalid data, on the voluntary basis, we finally recruited 50 undergraduates to participate in the study (18 males, 32 females; *M*_age_ = 21.92, *SD* = 1.20, ranging from 19.33 to 23.75). All participants’ nationality was Han and they assigned the informed content before the task. For their participation, they could choose to get 10 RMB cash or a small gift worth 10 RMB (following their preference).

### Materials and procedure

Participants took part in Study 1 in a group of 6 ~ 8. Upon arriving at the lab, they were told that they would complete an impression formation task, and then, we introduced the trait-rating task for them.

The trait-rating task consisted of two targets needing to be evaluated (junior leader and senior leader), and eight pairs of warmth traits (e.g., friendly vs. unfriendly) which were selected from previous research (Li et al., 2022). All 8 pairs of warmth traits in Study 1 were listed in Table 1. For each target, participants needed to mark a number on the 11-points scale (ranging from −5 to 5) to indicate which one of each pair of traits and to what extent it was suitable to represent the target. For example, about the target *senior leader* and the given trait-pair “friendly vs. unfriendly,” if one marked “-5” on the 11-points scale, it means that he/she thought that “unfriendly” was extremely suitable to represent the *senior leader*. In contrast, if one marked “5” on the 11-points scale, it means that he/she thought that “friendly” was extremely suitable to represent the *senior leader*. In this way, participants successively gave their ratings for the senior and junior HP targets on all eight pairs of warmth traits. The presentation order of two targets was counterbalanced across participants.

**Table 1:** Two evaluated targets and eight pairs of warmth traits used in the trait-rating task of Study 1.

Evaluated targets	Warmth traits	
	Positive	Negative
Senior leader	friendly	unfriendly
Junior leader	upright	cunning
	genuine	hypocritical
	enthusiastic	indifferent
	modest	supercilious
	easy-going	domineering
	amiable	rigid
	altruistic	self-serving

Following the trait-rating task, participants provided their demographic information (gender, age, and nationality). After that, we expressed our thanks to participants and answered their all questions about the study. Finally, we gave each participant a small gift or 10 RMB cash and guided them to leave the lab.

### Results

One male participant was dropped from data analysis because he misunderstood the instruction and marked two numbers for each trait-pair. So, a total of 49 participants were included in the final data analysis. Following previous research (Li et al., 2022; Tanjitpiyanond et al., 2022), we calculated the rating scores about senior and junior HP targets by separately averaging the scores on all trait-pairs for the two targets. As a result, the rating scores about senior and junior HP targets were separately generated. Because gender did not produce any significant effects, this variable was not further considered. In the current research, all data analyses aiming to explore possible gender effects were provided in supplementary materials. We conducted a paired-samples T test to examine whether participants indicated different rating scores about senior and junior targets. The results showed that participants indicated more positive evaluations for the senior target than for the junior target (*M*_senior_ = 1.54, *M*_junior_ = −0.01), *t*(48) = 6.17, *p* < 0.001, d = 0.84.

### Discussion

Consistent with previous research (Li et al., 2022), the results of Study 1 suggested that participants displayed more positive warmth perceptions for the senior HP target than for the junior HP target on the relative level. Thus, Study 1 provided initial evidence for our Hypothesis 1 on the explicit level. In the next Study 2, by employing the IAT, we sought to examine participants’ warmth perception about senior and junior targets on the implicit level so that we could provide converging evidence for our Hypothesis 1.

## Study 2

The aim of Study 2 was to examine participants’ warmth perception about senior and junior targets by using the IAT. In the IAT, we defined senior and junior HP labels as the target dimension, and defined positive and negative warmth traits as the attribute dimension. According to the results of Li et al.’ (2022) research (Study 3), on the relative level, senior HP individuals were considered to display positive warmth traits, while junior HP individuals were considered to display negative warmth traits. Therefore, in the IAT of Study 2, the combinations of “senior HP labels—positive warmth traits” and “junior HP labels—negative warmth traits” were defined as the compatible responses, while the combinations of “senior HP labels—negative warmth traits” and “junior HP labels—positive warmth traits” were defined as the incompatible responses. In the subsequent “Materials and procedure” section, we would provide more detailed introductions about the IAT.

### Participants and design

As in Study 1, the design of Study 2 was a single-factor within-subjects design with two levels (senior HP vs. junior HP). According to the calculation of G*Power 3.1 (Faul et al., 2009), at least 45 participants can meet the requirement of a presupposed effect size 0.5 and being significant at the 0.05 level. Considering possible invalid data, we finally recruited 48 undergraduates to take part in Study 2 (16 males, 34 females; *M*_age_ = 20.91, SD = 1.18, ranging from 19.17 to 23.5). Among 48 participants, 47 participants’ nationality was Han and 1 participant’s nationality was Hui.

### Materials and procedure

The procedure of Study 2 was identical to that of previous research by Li et al. (2022). Participants took part in the study in a group of 6 ~ 8. Upon arriving at the lab, they were told that they would complete a hand-eye coordination task (the IAT). If they agreed to continue with the task, they needed to sign the informed consent. Then, we gave participants a detailed introduction about the IAT.

We told participants that the so-called coordination task included seven parts. During each part, they needed to make responses by pressing the *E* or *I* key of the keyboard following the instructions presented on the screen. In Part 1, participants needed to classify 24 warmth traits into the “positive” or “negative” category by pressing the *E* key (for positive traits) or the *I* key (for negative traits). When they pressed a wrong key, the screen would present the feedback “wrong response.” These 24 warmth traits consisted of all 16 warmth traits used in Study 1 and the selected 8 traits from them in a pseudo-random way (4 positive warmth traits and 4 negative warmth traits). That means, there were 4 positive warmth traits and 4 negative warmth traits to present for two times in Part 1.

In Part 2, participants needed to classify 24 HP labels into the “senior HP” or “junior HP” category by pressing *E* key (for senior HP labels) or the *I* key (for junior HP labels). The 24 HP labels actually were 8 HP labels presented three times which were identical to those of the research by Li et al. (2022), and such HP labels were also listed as follow: minister, director general, senior official, board chairman, section chief, workshop manager, community director, and village secretary.

**Figure 1:**
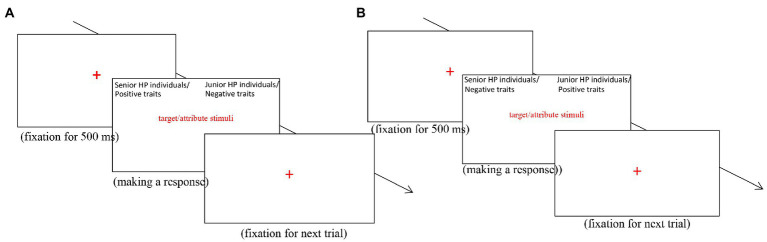
The trial structure in Part 3 and 4 of the IAT **(A)** and the trial structure in Part 6 and 7 of the IAT **(B)** were schematically illustrated.

Part 3 and 4 were the compatible combination-response parts in which participants were instructed to press the *E* key when a senior HP label or a positive warmth trait was presented on the screen, and press the *I* key when a junior HP label or a negative warmth trait was presented on the screen (see Figure 1). In line with previous research (Carlsson and Björklund, 2010), the only difference between the two parts was that Part 3 included 24 trials while Part 4 included 48 trials (the 16 traits in Part 1 and the 8 HP labels in Part 2 were both presented for two times).

In Part 5, participants made the key-press in contrary to Part 1. That is, they needed to press the *E* key when a negative trait was presented on the screen and press the *I* key when a positive warmth trait was presented on the screen. To assure that participants were familiar with the new key-press, Part 5 included 48 trials.

Part 6 and 7 were the incompatible combination-response parts. In both parts, participants were instructed to press the *E* key when a senior HP label or a negative warmth trait was presented on the screen, and press the *I* key when a junior HP label or a positive warmth trait was presented on the screen (see Figure 1). The two parts were identical except that Part 6 included 24 trials and Part 7 included 48 trials (the 16 traits in Part 1 and the 8 HP labels in Part 2 were both presented for two times). The whole IAT took about 15 ~ 17 min.

After the IAT, participants provided their demographic information (gender, age, and nationality), and then received 10 RMB or an equivalent gift. During the debriefing, no participant expressed suspicions about the purpose of the study. Finally, they were guided to leave the lab.

### Results

Following the recommendation by Greenwald et al. (2003), we calculated the D value for each participant. All trials from the Part 3, 4, 6, and 7 were included in the data analysis. Those trials lower than 300 ms or longer than 10,000 ms were regarded as invalid data, and thus removed from the data analysis. Following this, the mean difference in response times for the trials in Part 3 and 6 was calculated for each participant. The calculated mean difference was then divided by the participant’s inclusive standard deviation in response times for these two parts. By this way, the mean difference of each participant in response times for Part 4 and 7 also was calculated, and then divided by the participant’s inclusive standard deviation in Part 4 and 7. Then, these two figures were averaged, which generated a D value. The D value is the individual’s standardized score of the IAT, with a positive value reflecting stronger association strength for the compatible combination-responses, and a negative value reflecting stronger association strength for the incompatible combination-responses.

Preliminary analyses showed that gender did not yield any significant effects, so this variable was not included in the subsequent analyses. To examine whether participants implicitly associated positive warmth traits with senior HP labels and associated negative warmth traits with junior HP labels on the relative level, we conducted a one-sample *T* test to examine whether the generated D values were significantly greater than 0. The result showed that the average D value was significantly greater than 0 (*M* = 0.75), *t*(49) = 14.66, *p* < 0.001, d = 2.07, suggesting that participants held implicit power stereotypes that they tended to associate senior HP targets with positive warmth traits and associate junior HP targets with negative warmth traits.

### Discussion

In Study 2, we used the IAT to assess participants’ implicit power stereotypes about HP individuals. Corroborating the findings of Study 1, the results suggested that even on the implicit level, participants were also inclined to perceive senior HP targets as displaying more warmth than junior HP targets. So far, by applying two different paradigms to examine the power stereotypes on the explicit and implicit levels, we conceptually replicated Li et al.’ (2022) work and also provided convincing evidence for our Hypothesis 1 that on the relative level, participants showed more positive warmth perceptions toward senior HP targets than toward junior HP targets. In the next Study 3, we would further examine whether this subtle difference about the warmth perception toward senior and junior HP individuals would have an influence on people’s interpretation about behaviors. More concretely, we aimed to examine how participants’ power stereotypes about senior and junior HP individuals affected the occurrence of STI.

## Study 3

As documented above, in Study 3, we mainly explored whether the perceived warmth difference about senior and junior HP individuals would have an influence on the occurrence of STI. Previous two studies revealed that participants tended to associate senior HP individuals with positive warmth traits and associate junior LP individuals with negative warmth traits. Based on this, we reasoned that when people attempted to interpret trait-implying behaviors, the presentation of senior HP actors would make them more likely to make STI from behaviors implying positive warmth traits than from behaviors implying negative warmth traits, whereas the presentation of junior HP actors would make perceivers more likely to make STI from behaviors implying negative warmth traits than from behaviors implying positive warmth traits. In short, the power stereotypes induced by senior and junior HP labels would facilitate the STI from stereotype-consistent behaviors in comparison with stereotype-inconsistent behaviors (Hypothesis 2). In Study 3, we would conduct an empirical test for this hypothesis.

### Participants and design

Study 3 was a 2 (actor power: senior vs. junior) × 2 (trait valence: positive vs. negative) with-subjects design. According to the calculation of G*Power 3.1 (Faul et al., 2009), a presupposed medium effect size (*f* = 0.25) and being significant at the 0.05 level needed at least 36 participants. Considering possible invalid data, we finally recruited 40 participants to participate in this study (17 males, 23 females; *M*_age_ = 20.26, SD = 0.46, ranging from 19.33 to 21.33). All participants’ nationality was Han. For their participation, they can receive 10 RMB or an equivalent gift.

### Materials

#### The probe recognition paradigm

The probe recognition paradigm, initially developed by McKoon and Ratcliff (1986), would be used to test our Hypothesis 2. In the paradigm, researchers commonly present a series of trait-implying behavioral sentences *via* the computer and each sentence will stay on the screen for a very short time. Following each sentence, a probe word will appear and participants need to press specific keys to indicate whether the probe has been included in the paired behavioral sentence. When the probe recognition paradigm is applied to examine the effects of stereotypes on STI, researchers generally assign different social labels for actors of trait-implying behaviors to generate stereotype-consistent and stereotype-inconsistent behavioral sentences. For example, when the label “professor” was denoted as the subject of the sentence “The X wins the science quiz,” a stereotype-consistent sentence was created (Wigboldus et al., 2003). In contrast, when the label “garbage man” was denoted as the subject of the above sentence, a stereotype-inconsistent sentence was created. As a result, participants can readily indicate a correct “No” response when the probe “smart” appeared following the sentence “The garbage man wins the science quiz,” but they often need longer time to indicate the “No” response when the probe “smart” appeared following the sentence “The professor wins the science quiz.” By comparing the RTs for stereotype-consistent sentences with the RTs for stereotype-inconsistent sentences (longer RTs for stereotype-consistent than stereotype-inconsistent sentences in general), the effects of stereotypes on STI thus can be assessed. The procedure of the probe recognition paradigm was schematically presented in Figure 1.

#### Stereotype-consistent and -inconsistent trials

We developed 12 stereotype-consistent trials and 12 stereotype-inconsistent trials following the steps as below. Firstly, 6 positive warmth traits and 6 negative warmth traits were selected from Study 2. Then, for each trait, we developed two behavioral sentences implying the trait and the subject of each sentence was temporarily denoted as “X.” The X was regarded as a leader whose identity was not specified when developing trait-implying sentences. Additionally, all behaviors were limited to the communications between HP and LP individuals.

Totally, 24 trait-implying behavioral sentences were generated and all trait-implying sentences were provided in Table 2. In a pretest, we asked 35 undergraduates to indicate to what extent each sentence imply the corresponding trait on the 5-points scale (1 = *not at all*, 5 = *completely*). The results showed that such trait-implying sentences significantly implied the corresponding trait, *M* = 4.14, ranging from 3.94 to 4.31. For the 12 positive trait-implying sentences, half of them were denoted as senior HP actors to form 6 stereotype-consistent trials about senior HP individuals (e.g., The secretary spilled water on the book, the general manager told her that “took it easy, took your time”), and the other half were denoted as junior HP actors to form stereotype-inconsistent trials about junior HP individuals (e.g., The mission suffered a second setback due to a clerk’s negligence, and the department manager patted the clerk on the shoulder as a reminder). In a similar way, for the other 12 negative trait-implying sentences, half of them were denoted as junior HP actors to form stereotype-consistent trials about junior HP individuals (e.g., The workshop manager told her subordinates that “do your job well if you want, otherwise leave right now”), and the other half were denoted as senior HP actors to form stereotype-inconsistent trials about senior HP individuals (e.g., The chairman shouted to subordinates that “I’m your leader and you must comply with me regardless of your intentions”). Totally, 12 stereotype-consistent (6 senior HP stereotype-consistent trials and 6 junior HP stereotype-inconsistent trials) and 12 stereotype-inconsistent trials were created (6 senior HP stereotype-consistent trials and 6 junior HP stereotype-inconsistent trials).

**Table 2:** The trait-implying sentences used in Study 3 and to what extent each sentence implied the corresponding trait.

Traits	Behavioral sentences	*M* (implying the trait)
friendly	Given the cold weather, the X offered a cup of hot coffee for the visitor.	4.14
	When taking a group photo, the X gave an active hug for subordinates around them.	4.14
unfriendly	The X refused to listen the explanation that why the subordinate has been late for the meeting.	4.14
	When communicating with subordinates at the meeting, the X always keeps a straight face.	4.11
upright	The X rejected the proposition of promoting the development of the organization at the cost of staff welfare.	4.23
	The X rejected the proposition from a friend because it in nature “harmed others to benefit himself.”	4.14
cunning	To improve work efficiency, the X in private gathered information about subordinates’ attitudes at work.	4.20
	To maintain her status, the X made another financial statement exclusively for the audit supervision.	4.14
genuine	At the morning meeting, the X introduced the encountering financial pressure for the team in a detailed way.	4.17
	After a series of failures, the X told everyone that there may be a fatal flaw existing in the original project.	4.17
hypocritical	The X alleged that he would treat everyone in the same way, but he allocated the best resources for himself.	4.26
	Although the X realized that the job was quite boring, she still told subordinates that she loved it very much.	4.09
enthusiastic	When subordinates faced technical difficulties, the X took the initiative to offer several solutions.	4.17
	When hearing that the secretary had been ill, the X asked whether she needed to take a few more days off.	4.00
indifferent	The X deleted the email halfway through it in which subordinates complained that they are overworked.	4.06
	About the low salary of subordinates, the X told himself that “it’s not my business.”	4.09
modest	At the meeting, the X said that the success of the project mainly depended on everybody’s efforts, rather than his contribution.	4.14
	As a technical expert, the X answered the questions one by one which were proposed by some newcomers.	3.94
supercilious	The X told her subordinates that “do your job well if you want, otherwise leave right now.”	4.11
	The X shouted to subordinates that “I’m your leader and you must comply with me regardless of your intentions.”	4.03
amiable	The mission suffered a second setback due to a clerk’s negligence, and the X patted the clerk on the shoulder as a reminder.	4.26
	The secretary spilled water on the book, the X told her that “take it easy, take your time.”	4.11
rigid	Due to one minute late, the X sharply berated the clerk for being late in front of all subordinates.	4.20
	Due to failing to reach his expectation, the X asked subordinates to modify the report until the midnight.	4.31

*M* = mean value.

#### Filler trials

Considering that both stereotype-consistent and stereotype-inconsistent trials would induce the “No” response, we developed 12 filler trials in which the probe was included in the preceding sentence and thus would induce the “Yes” response (they were provided in supplementary materials). Among 12 filler behavioral sentences, half of them were denoted as senior HP actors and the other half were denoted as junior HP actors. All filler trials were provided in supplementary materials.

### Procedure

**Figure 2:**
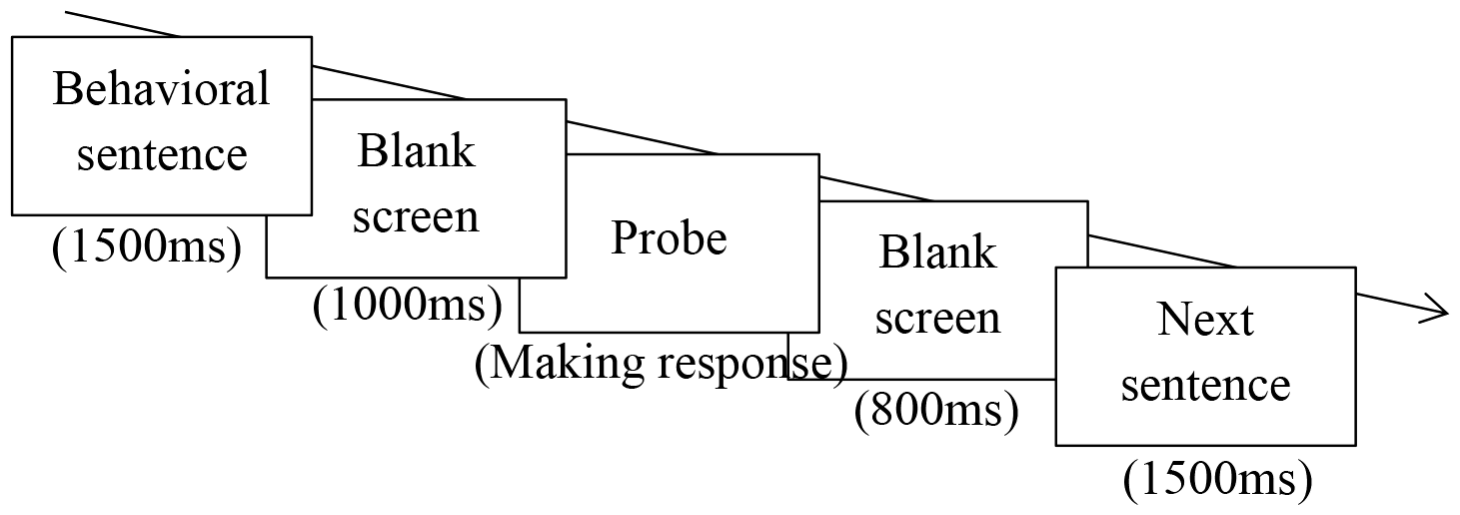
Trial structure of the probe recognition paradigm was illuminated schematically.

Participants were invited to participate in this experiment in a group of 6 ~ 8. When arriving at the lab, they learned about the academic purpose of the study and then assigned the informed content on the voluntary basis. Following that, we ostensibly told participants that they would complete a so-called memory task (the probe recognition task). In the task, we presented a series of sentences *via* the computer and each sentence stayed on the screen for 1,500 milliseconds (ms). Following each sentence, a blank screen appeared for 1,000 ms. After that, a probe appeared on the screen until participants pressed the corresponding key (see Figure 2). After participants indicated their response, there was a blank screen lasting for 800 ms, and then, the next sentence appeared on the screen. All stimuli were presented *via* E-prime 3.0 (Psychology Software Tools, Inc.).

After completing the probe recognition task, participants provided their demographic information (gender, age, and nationality). During the debriefing, we responded to participants’ any questions about the experiment. Finally, we offered rewards for participants and guided them to leave the lab.

### Results

#### Preliminary analyses

As did in previous research (Ramos et al., 2012), the RTs of wrong responses and the RTs longer than 3,000 ms were deleted from our database (5.93%). The logic under this operation was that the probe recognition paradigm was quite easy for undergraduates, so, if one participant indicated his response with RTs longer than 3,000 ms, there actually was a high probability that the participant did not focus his/her attention on the task at that time. The deleted data was replaced with the corresponding series mean. Then, we computed the RTs for stereotype-consistent trials by separately averaging participants’ RTs for the “senior HP actor—positive warmth trait” trials and the “junior HP actor—negative warmth trait” trials. Similarly, we computed the RTs for stereotype-inconsistent trials by separately averaging participants’ RTs for the “senior HP actor—negative warmth trait” trials and the “junior HP actor—positive warmth trait” trials. The calculated mean values were provided in Table 3. Preliminary analyses did not revealed any significant effects relevant to gender, so this factor was not further considered.

**Table 3:** Participants’ mean value and standard deviation in each condition across Study 3, 4 and 5 (*M* ± SD).

Dependent variable index		Study 3			
		Senior HP actor		Junior HP actor	
		Positive	Negative	Positive	Negative
RTs		802.35 ± 246.35	725.25 ± 175.15	743.17 ± 206.11	781.84 ± 224.83
		Study 4			
		**Senior HP actor**		**Junior HP actor**	
		**Positive**	**Negative**	**Positive**	**Negative**
ERs		0.46 ± 0.28	0.31 ± 0.35	0.33 ± 0.24	0.44 ± 0.32
		Study 5			
		**Senior HP actor**		**Junior HP actor**	
		**Positive**	**Negative**	**Positive**	**Negative**
ERs	Chinese culture	0.43 ± 0.28	0.29 ± 0.34	0.32 ± 0.24	0.41 ± 0.30
	Western culture	0.30 ± 0.26	0.45 ± 0.24	0.34 ± 0.22	0.44 ± 0.30

*M* = mean value, SD = standard deviation, RTs = reaction times, ERs = error rates.

#### The effects of stereotypes on STI

To examine whether participants’ stereotypes about senior and junior HP individuals would produce an influence on the STI, we submitted the calculated RTs into a 2 (actor power: senior vs. junior) × 2 (trait valence: positive vs. negative) repeated-measures analysis of variance (ANOVA). The analysis revealed a significant main effect of trait valence, *F*(1, 39) = 4.36, *p* = 0.04, partial *η*^2^ = 0.10, as participants spent slightly longer time responding to positive traits than responding to negative traits (*M*_positive_ = 772.76, *M*_negative_ = 753.54). However, this significant main effect was qualified by a quite significant interaction effect between actor power and trait valence, *F*(1, 39) = 12.46, *p* = 0.001, partial *η*^2^ = 0.24. The subsequent simple-effects analyses revealed that participants displayed longer time for positive warmth traits than for negative warmth traits when behavioral actors were senior HP individuals, *t*(39) = 3.95, *p* < 0.001, d = 0.36 (*M*_positive_ = 802.35, *M*_negative_ = 725.25, respectively), whereas an opposite pattern emerged when behavioral actors were junior HP individuals—participants displayed longer time for negative warmth traits than for positive warmth traits, *t*(39) = 2.14, *p* = 0.04, *d* = 0.18 (*M*_positive_ = 743.17, *M*_negative_ = 781.84, respectively).

### Discussion

In Study 3, we applied the probe recognition paradigm to examine the effects of power stereotypes on STI. Consistent with our expectation, the results demonstrated that participants were more likely to make STI from those behaviors implying positive warmth traits than from those behaviors implying negative warmth traits when behavioral actors were senior HP individuals. In contrast, participants were more likely to make STI from those behaviors implying negative warmth traits than from those behaviors implying positive traits when behavioral actors were junior HP individuals. Such results provided initial support for our Hypothesis 2. In the next Study 4, we aimed to use another paradigm—the false recognition paradigm—to further confirm the robustness of the effects of power stereotypes on STI.

## Study 4

Study 4 was conducted to achieve two primary goals. The first goal, as mentioned above, was to confirm the effects of power stereotypes on STI by using a different paradigm—the modified false recognition paradigm, which has been widely used to detect the occurrence of STI (for a review, see Uleman et al., 2012). The detailed introductions about the modified false recognition paradigm were presented in the “Materials” section. The second goal of Study 4 was to empirically test our Hypothesis 3 that participants’ perception about social responsibility of HP individuals would account for the effects of power stereotypes on STI. To achieve this goal, we would assess participants’ perception about social responsibility of HP individuals using a specific scale, and then examined the prediction effect of perceived social responsibility on the power stereotype effects in STI. We anticipated that the more social responsibility participants perceived about senior HP individuals (compared to junior HP individuals), the stronger power stereotype effects they showed in STI.

### Participants and design

Similar to that of Study 3, the design of Study 4 also was a 2 (power label: senior vs. junior) × 2 (trait valence: positive vs. negative) with-subjects design. We determined the sample size of Study 4 based on the calculation of G*Power 3.1, which suggested that we needed at least 36 participants to meet the demands of the effect size 0.25 and being significant at the 0.05 level. However, considering that we would conduct a regression analysis to test our Hypothesis 3 in which power stereotype effects in STI would be regressed on perceived social responsibility and 36 participants may be not sufficient to afford the analysis. Given that, following previous research (Wang and Yang, 2017; Li et al., 2022), we finally recruited 100 participants in this study (35 males, 65 females; *M*_age_ = 21.18, *SD* = 0.49, ranging from 20.17 to 22.75). All participants’ nationality was Han. This sample size was determined before we did any data analysis. For their participation, participants can receive 10 RMB or an equivalent gift.

### Materials

#### The modified false recognition paradigm

The classic false recognition paradigm includes two phases—study phase and recognition phase (Todorov and Uleman, 2002, 2003). In the study phase, participants are presented with a series of photo-behavioral sentence pairs (one sentence per photo) and they are instructed to try their best to memorize such photo-behavioral sentence pairs. In the subsequent recognition phase, participants are presented with a series of photo-trait pairs. For each photo-trait pair, participants need to press specific keys to indicate whether the trait had been included in the behavioral sentence paired with the photo in the study phase. The core assumption of the false recognition paradigm is, if participants make STI from one trait-implying sentence in the study phase, in the subsequent recognition phase, they should be more likely to display false recognition when the trait implied by the behavior is paired with the actor’s photo than when the trait is paired with some other photo (Todorov and Uleman, 2002).

**Figure 3:**
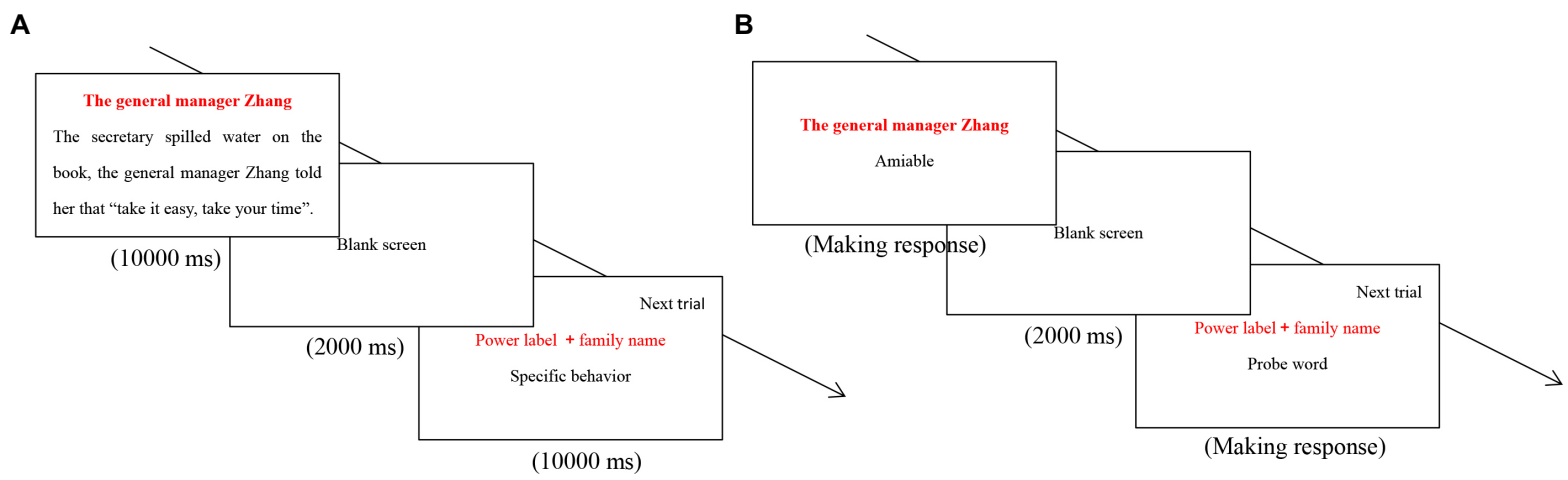
Trial structures of the study phase **(A)** and recognition phase **(B)** in the modified false recognition paradigm.

In the study phase of Study 4, we also presented a series of behavioral sentences for participants. However, different from the classic false recognition paradigm, each behavioral sentence in Study 4 was paired with the actor’s power label rather than his/her photo. More concretely, each “power label—behavioral sentence” pair was presented on two separate lines—the power label of the actor on the first line and the behavioral sentence on the second line (see Figure 3). To increase the salience of power label, each actor’s power label was colored by red font. Additionally, we told participants that, for the purpose of effectively remembering such sentences, they should simultaneously pay attention to actors’ power label and their behaviors. In the recognition phase, we successively presented a series of “actor-trait” pairs in which the actor was presented in the form of “family name + power label” (e.g., The General Manager Zhang) and the trait had been implied by the sentence paired with the actor in the study phase (see Figure 3). According to the results of Study 3, in Study 4, we predicted that participants may be more likely to make false recognition for positive warmth traits than for negative warmth traits when the actors of behaviors were denoted as senior HP labels, whereas they may be more likely to make false recognition for negative warmth traits than for positive warmth traits when the actors of behaviors were denoted as junior HP labels. In simple terms, we anticipated that compared to stereotype-inconsistent pairs, participants were more likely to make false recognition for stereotype-consistent pairs.

#### Experimental and filler trials

In the modified false recognition paradigm, the study phase included 24 “power label-behavioral sentence” pairs, 6 stereotype-consistent pairs (3 senior HP stereotype-consistent pairs and 3 junior HP stereotype-consistent pairs), 6 stereotype-inconsistent pairs (3 senior HP stereotype-inconsistent pairs and 3 junior HP stereotype-inconsistent pairs), and 12 filler pairs (HP labels were paired with stereotype-irrelevant sentences). All behavioral sentences were selected from the trait-implying sentences of Study 3, which were provided in supplementary materials. For each “power label-behavioral sentence” pair, they would stay on the screen for 10,000 ms. After that, a blank screen would be presented for 2,000 ms until the next “power label-behavioral sentence” pair appeared on the screen (see Figure 3).

In the recognition phase, 24 “power label-probe word” pairs would be successively presented on the screen and participants needed to press the *A* (the Yes response) or the *L* (the No response) key to indicate whether the probe word had been included in the sentence paired with the power label in the study phase. A 2,000 ms blank screen followed the response and then the next “power label-probe word” would appear on the screen.

#### The measure of perceived social responsibility

In Study 4, the modified Confucianism Identification Scale developed by Li et al. (2022) was used to measure participants’ perception about the social responsibility of senior and junior HP individuals. The Confucianism Identification Scale is initially developed to measure participants’ perception about the social responsibility of HP individuals in comparison to LP individuals, and the scale contains three items: (a) in my view, individuals with greater power should display more benevolence toward others; (b) if one person possesses social power, he/she should pay more attention to the livelihood of ordinary people; (c) and those people with great social power generally have a high degree of social responsibility rather than only pursue their own enjoyment. The scale has displayed a good suitability in Confucian and non-Confucian cultures (Li et al., 2022).

In the current research, we modified the comparison between HP and LP individuals into the comparison between senior and junior HP individuals. Specifically, we added the adverbial clause of condition “compared with junior HP individuals, senior HP individuals …” in the beginning of each item (e.g., in my view, compared with junior senior HP individuals, senior HP individuals should display more benevolence toward others). Before participants began to fill out the scale, we told participants that junior HP individuals refer to those HP individuals who are primarily responsible for directly managing ordinary people, such as workshop manager or community director, and senior HP individuals refer to those HP individuals who are primarily responsible for managing junior HP individuals, such as general manager or senior official. After that, participants indicated to what extent they agreed with each item on the 5-points scale (1 = *strongly disagree*, 5 = *strongly agree*). We assessed participants’ perception about the social responsibility of senior and junior HP individuals by summarizing the scores across three items and a higher aggregate score indicates a higher expectation that senior HP individuals have stronger social responsibility than junior HP individuals. In the current research, the Cronbach’s *α* of the scale was 0.72.

### Procedure

The experiment was conducted in the lab. At the appointed time, participants arrived at the lab in a group of 6 ~ 8. Upon arrival, we told participants that this study was an definitely academic survey about memory and impression formation, and all the data collected from the study was used only for academic purpose, but not for commercial purpose. If they were willing to continue the experiment, they needed to sign the informed content.

Then, we introduce the so-called memory task (the false recognition paradigm) for participants in a detailed way. In the study phase of the memory task, we told participants that they needed to try their best to memorize a series of sentences presented on the computer. In the recognition phase, we instructed participants to press the given keys to indicate whether the probe word had been included in the sentence presented in the study phase. During the both study and recognition phases, three exercise trials were provided to make participants familiar with the procedure.

Following the memory task, participants completed the measure of perceived social responsibility about senior and junior HP individuals, and provided their demographic information (gender, age, and nationality). During the subsequent debriefing, we answered all participants’ questions relevant to this study. Finally, they received their rewards and left the lab.

### Results

#### Preliminary analyses

Due to the unexpected power cut, the data from 6 participants had not been collected successfully (4 males, 2 females). As a result, a total of 94 participants were included in final data analyses. Overall, the average ERs of participants was 0.31, significantly lower than 0.5, *t*(93) = 11.97, *p* < 0.001, *d* = 1.23. Then, we calculated participants’ average ERs separately for the “senior HP label-positive trait” pairs (*M* = 0.46), the “senior HP label-negative trait” pairs (*M* = 0.31), the “junior HP label-positive trait” pairs (*M* = 0.33), and the “junior HP label-negative trait” pairs (*M* = 0.44). All average ERs were presented in Table 3. Preliminary analyses did not find any significant effects relevant to gender, this variable thus was dropped from subsequent data analyses.

#### The effects of power stereotypes on STI

To examine the effects of power stereotype on STI, we submitted participants’ ERs across four conditions into a 2 (power label: senior vs. junior) × 2 (trait valence: positive vs. negative) repeated-measures ANOVA. The results showed that the main effects of target power and trait valence were neither significant, *F*(l, 93) = 0.03, *p* = 0.87, partial *η*^2^ < 0.001, *F*(l, 93) = 0.44, *p* = 0.51, partial *η*^2^ = 0.005, respectively. However, the expected interaction between power label and trait valence was significant, *F*(l, 93) = 25.04, *p* < 0.001, partial *η*^2^ = 0.21. Simple-effects analyses showed that when the actors of behavioral sentences were denoted as senior HP labels, participants made more false recognition for the “senior HP label—positive trait” pairs than for the “senior HP label—negative trait” pairs, *t*(93) = 3.68, *p* < 0.001, *d* = 0.47; by contrast, participants made more false recognition for the “junior HP label—negative trait” pairs than for the “junior HP label—positive trait” pairs, *t*(93) = 2.57, *p* = 0.01, *d* = 0.40. That is to say, for the both “senior HP label—probe trait” and “junior HP label—probe trait” pairs, participants were more likely to make false recognition for the stereotype-consistent pairs than for the stereotype-inconsistent pairs.

#### The prediction effect of perceived social responsibility on power stereotype effects in STI

According to Hypothesis 3, participants’ perception about social responsibility of HP individuals accounted for the power stereotypes in STI. To test this hypothesis, we conducted a regression analysis in which power stereotype effects in STI were regressed on perceived social responsibility. Perceived social responsibility was calculated by summing the scores on all three items and power stereotype effects in STI were calculated by subtracting the ERs for stereotype-inconsistent pairs from the ERs for stereotype-consistent pairs. Both variables were standardized before entering the regression equation. The results showed that the prediction effect of perceived social responsibility was significant, standardized regression coefficient *β* = 0.35, *t*(92) = 3.63, *p* < 0.001. This result indicated that the more social responsibility participants perceived about senior HP individuals, the stronger stereotype effects they showed in STI, thus supporting Hypothesis 3.

### Discussion

In Study 4, we employed the modified false recognition paradigm to examine the effects of power stereotypes on STI. Consistent with the results of Study 3, we found that participants were more likely to draw STI from behavioral sentences implying positive warmth traits than from behavioral sentences implying negative warmth traits when the actors of behaviors were denoted as senior HP labels, whereas participants were more likely to draw STI from behavioral sentences implying negative warmth traits than from behavioral sentences implying positive warmth traits when the actors were denoted as junior HP labels. Combining the results of Study 3 with the results of Study 4, we have provided converging evidence for Hypothesis 2. In Study 4, we also found that those participants who were inclined to posit that senior HP individuals showed greater social responsibility than junior HP individuals displayed stronger power stereotype effects in STI, thus initially supporting our Hypothesis 3 that perceived social responsibility about HP individuals accounted for the power stereotype effects in STI.

In the next Study 5, we aimed to further explore whether the observed stereotype effects in Study 3 and 4 would exist exclusively in Confucian culture. According to our Hypothesis 4, we expected that the effects of power stereotypes on STI would be observed in Confucian culture but not in non-Confucian culture.

## Study 5

The primary aim of Study 5 was to test whether the observed power stereotype effects in STI were Confucian culture-specific, so that we can provide empirical evidence for our Hypothesis 4. To this end, we conducted an online cross-cultural study *via* the Credamo,^1^ which is a professional data collection platform widely used by psychologists in China-Mainland. In this cross-cultural study, we utilized the modified false recognition paradigm to compare whether there were any differences about the effects of power stereotypes on STI between Confucian and non-Confucian cultures. Based on Hypothesis 4, we postulated that the observed power stereotype effects in previous two studies would only emerge in Confucian culture but not in non-Confucian culture. Additionally, in Study 5, we again examined the relationship between perceived social responsibility and power stereotype effects in STI. We reasoned that, if power stereotype effects in STI were Confucian culture-specific, perceived social responsibility would yield a significant prediction effect on power stereotype effects in Confucian culture rather than in non-Confucian culture.

### Participants and design

The design of Study 5 was a 2 (culture: Confucian culture vs. non-Confucian culture) × 2 (power label: senior vs. junior) × 2 (trait valence: positive vs. negative) mixed-measures design, with culture as the between-subjects variable. The sample size was determined by the calculation of G*Power 3.1 which showed that the effect size 0.25 and the significant level 0.05 required at least 132 participants (Faul et al., 2009). *Via* the Credamo platform, we finally recruited 140 participants to participate in this study. Considering the potential effects of age on individuals’ memory and STI (Zacks et al., 2000; Shimizu, 2012), we predetermined that only participants who were born between 1985 and 2002 were eligible to take part in this study, because individuals aging from 20 to 40 years old were considered to have a relatively good memory (Lin, 2018). Following Li et al.’ (2022) recommendation, half participants were recruited from China, a typically Confucian society (26 males, 44 females; 68 Han nationality, 2 Hui nationality; *M*_age_ = 25.43 years old, ranging from 19.83 to 37.33), and the other half were recruited from America, a typically non-Confucian society (31 males, 39 females; 55 whites, 10 blacks, and 5 Asians; *M*_age_ = 26.58 years old, ranging from 19.67 to 37.25). All Chinese participants needed to be fluent in Chinese, and all American participants needed to be fluent in English and were not first-generation immigrants.

### Materials and procedure

The materials and procedure of Study 5 were identical to those of Study 4 except that the materials of Study 5 were presented online *via* the Credamo. All materials included two equivalent versions—Chinese version (for Chinese participants) and English version (for American participants). To confirm the equivalence between the two versions, the instructions, behavioral sentences, and probes in the Chinese version were translated into English following the bask-translation procedure (Brislin, 1980). Firstly, the original Chinese version of the above experimental materials was translated into the English version by a psychology Ph.D. student who was fluent in Chinese and English. Then, the developed English version was translated back into the Chinese version by another Ph.D. student who was also fluent in Chinese and English. Finally, the new Chinese version was compared with the original version, and any discrepancies arising from the back-translation process were then adjusted. It needed to be explained that the Confucianism Identification Scale was not included in the back-translation procedure, because the Chinese and English version of the scale have been developed in previous research (Li et al., 2022). The Cronbach’s α of the Chinese version was 0.71, and the Cronbach’s α of the English version was 0.69.

Before the formal task, participants needed to learn about the academic and non-commercial purpose of the research, and then, they would complete the so-called memory task (the false recognition task), which was identical to that of Study 4. After the memory task, they reported their perception about the social responsibility of senior HP individuals on the 5-points scale (1 = *strongly disagree*, 5 = *strongly agree*). As did in Study 4, we provided explanations about senior and junior HP individuals in the beginning section of the scale. Besides, they also needed to provide their demographic information (gender, age, and nationality). After completing all tasks, they would receive their rewards (10 RMB for Chinese participants, 2 USD for American participants).

### Results

#### Preliminary analyses

The data of one participant failed to be collected successfully due to unknown reasons. Thus, a total of 139 participants were included in our final data analysis. Overall, the average ERs of participants was 0.32, which was significantly lower than 0.5, *t*(138) = 15.78, *p* < 0.001, *d* = 1.11. As did in Study 4, we calculated participants’ average ERs separately for the “senior HP label-positive trait” pairs, the “senior HP label-negative trait” pairs, the “junior HP label-positive trait” pairs, and the “junior HP label-negative trait” pairs. The average ERs under each condition was presented in Table 3. Preliminary analyses did not reveal any meaningful effects relevant to gender, this variable thus was not further considered.

#### Power stereotype effects in STI across cultures

To examine whether power stereotypes in STI varied with specific cultural backgrounds, we conducted a 2 (culture: Confucian culture vs. non-Confucian culture) × 2 (power label: senior vs. junior) × 2 (trait valence: positive vs. negative) mixed-measures ANOVA. The analysis revealed a significant main effect of trait valence, *F*(1, 137) = 4.66, *p* = 0.03, partial *η*^2^ = 0.03. Besides, trait valence also produced a significant interaction effect with culture background, *F*(1, 137) = 12.75, *p* < 0.001, partial *η*^2^ = 0.09, and a significant interaction effect with power label, *F*(1, 137) = 6.55, *p* = 0.012, partial *η*^2^ = 0.05.

The above significant main and interaction effects were qualified by a significant three-way interaction among cultural background, power label, and trait valence. Simple-effects analyses showed that for Chinese participants, there was a significant interaction effect between power label and trait valence, *F*(1, 69) = 21.76, *p* < 0.001, partial *η*^2^ = 0.24, while there was only a significant main effect of trait valence for American participants, *F*(1, 68) = 20.69, *p* < 0.001, partial *η*^2^ = 0.23. Further analyses showed that, as shown in Table 3, in the recognition phase, Chinese participants made more false recognition for the “actor—positive warmth trait” pairs when actors were denoted as senior HP labels, *t*(69) = 3.29, *p* = 0.002, *d* = 0.47, but made more false recognition for the “actor—negative warmth trait” pairs when actors were denoted as junior HP labels, *t*(69) = 2.33, *p* = 0.023, *d* = 0.34. In contrast, American participants made more false recognition for the “actor—negative warmth trait” pairs regardless of the paired power labels, *t*(68) = 3.88, *p* = 0.05, *t*(68) = 2.40, *p* = 0.02, respectively.

#### The prediction effect of perceived social responsibility on the power stereotype effects in STI

To examine the prediction effect of perceived social responsibility on the power stereotype effects in STI across Confucian and non-Confucian cultures, as did in Study 4, we firstly calculated participants’ perceived social responsibility (*M* = 9.54 among Chinese participants, and *M* = 8.68 among American participants) and power stereotype effects in STI, and then performed a regression analysis separately for Chinese and American participants in which power stereotype effects in STI were regressed on perceived social responsibility. The results showed that there was a significant prediction effect of perceived social responsibility on power stereotype effects in STI for Chinese participants, *β* = 0.31, *t*(68) = 2.64, *p* = 0.01, whereas the prediction effect of perceived social responsibility on power stereotype effects in STI was not significant for American participants, *β* = 0.12, *t*(67) = 1.00, *p* = 0.32.

### Discussion

Using the modified false recognition paradigm, we conducted an online cross-cultural study to explore whether the power stereotype effects in STI were culture-specific. Supporting our Hypothesis 4, the effects of power stereotypes on STI were culture-specific—Chinese participants made stronger STI for behaviors implying positive warmth traits when actors were denoted as senior HP labels, but made more stronger STI for behaviors implying negative warmth traits when actors were denoted as junior HP labels; by contrast, American participants made stronger STI from behaviors implying negative warmth traits regardless of actors’ power labels. Additionally, in Study 5, we also found that perceived social responsibility had a significant prediction effect on power stereotype effects in STI under Chinese culture but not under Western culture, which not only provided converging evidence for Hypothesis 3 that Chinese participants’ greater expectation about the social responsibility of senior HP individuals (in comparison to junior HP individuals) accounted for the observed power stereotype effects in STI, but also further confirmed the culture-specific characteristic of the effects.

## General discussion

In the current research, five studies were conducted to examine the warmth perception difference between senior and junior HP individuals and how this difference affected the occurrence of STI. The results of Study 1 and 2 showed that participants in Confucian culture tended to perceive junior HP individuals as negative on the warmth dimension, but perceive senior HP individuals as positive on the warmth dimension. The following Study 3 and 4 revealed that such different warmth perceptions exerted an influence on the occurrence of STI—participants tended to make STI from behaviors implying negative warmth traits when behavioral actors were junior HP individuals while participants tended to make STI from behaviors implying positive warmth traits when behavioral actors were senior HP individuals. Study 4 also revealed that perceived social responsibility accounted for the power stereotype effects in STI. That is, the more social responsibility participants perceived about senior HP individuals (compared to junior HP individuals), the stronger power stereotype effects they showed in STI. In final Study 5, the different power stereotype effects in STI induced by senior and junior HP actors were only observed in Confucian culture, but not in non-Confucian culture. Additionally, the predicted effect of perceived social responsibility on power stereotype effects in STI was also significant only in Confucian culture.

### Implications for power stereotypes and STI

Consistent with Li et al.’ (2022) research, the results of Study 1 and 2 suggested that participants tended to associate senior HP individuals with positive warmth traits in comparison with junior HP individuals. Beyond prior research, the current research further found that such relatively positive evaluations for senior HP individuals could unconsciously affect the interpretation for others’ behaviors. In concrete terms, Study 3 and 4 showed that when behavioral actors were senior HP individuals, participants were more likely to make STI from trait-implying behaviors implying positive warmth traits than those implying negative warmth traits. Considering that STI has been evidenced to be an automatic attribution process in nature (Carlston and Skowronski, 2005; Crawford et al., 2007), the results of Study 3 and 4 thus firstly demonstrated that participants’ positive warmth perception for senior HP individuals could exert an influence on their interpretation and prediction for power-holders’ behaviors in an unobtrusive way. On the broad level, our results suggest power stereotypes can be regarded as an ideological tool serving for maintaining the power hierarchy (Magee and Galinsky, 2008). We can imagine that when an employee suffers from unfair treatments from his department manager, he will be less likely to immediately take action against the organization if he holds the belief that his general manager will display higher interpersonal warmth and solve his problem fairly. Moreover, according to our findings, even for the same behavior, employees seemingly are more likely to perceive interpersonal warmth from the general manager than the department manager. Under this vein, power stereotypes and their influences in STI obviously will benefit for the stability of power hierarchy in the organization or in the society.

The current research also carried implications for improving ecological validity of the research concerning stereotypes and STI. In the seminal work by Wigboldus et al. (2003), researchers have commented that STI should not be investigated “in a social vacuum,” because in real life, we know much more about an actor than just his or her behaviors. Supporting this viewpoint, the subsequent research demonstrated that some salient features of actors could activate the corresponding stereotypes, and the activated stereotypes further facilitated the STI from stereotype-consistent behaviors and inhibited the STI from stereotype-inconsistent behaviors (Wigboldus et al., 2003, 2004; Yan et al., 2012; Wang and Yang, 2017). In the past decade, however, researchers pay much attention to some “visible features” of actors when investigating the influences of stereotypes on STI, such as skin color, gender, and age (Wigboldus et al., 2003, 2004; Yan et al., 2012). As a consequence, the “invisible features” of actors are relatively ignored, such as actors’ power involved in the current research. Indeed, power label is a very important social label in daily life when we need to introduce someone in several short sentences or when we need to learn about someone under a situation in which a limited amount of information is provided (Magee and Galinsky, 2008). Power is so important that people can accurately figure out one’s position in the power hierarchy only based on some subtle clues (Mast and Hall, 2004; Carney et al., 2005). In addition to power, occupation is another important but invisible social label for one person (Henry et al., 2021), and future research can attempt to explore the possible effects of actors’ occupation on STI. Additionally, to mirror real life to a greater extent, exploring the interaction effects of actors’ power and occupation on STI may also be necessary. After all, everyone has more than one identity in real life (Sinclair et al., 2006), and more importantly, there is a high probability that the leader from a college and the leader from a company will invoke quite different stereotypes even though they both belong to HP individuals.

### Implications for the SCM

The current research enriched the understanding about cultural universality and specificity of the SCM. The SCM posits that warmth and competence are the key dimensions underlying judgments about social groups, and individuals with high social status are perceived as positive on the competence dimension and negative on the warmth dimension, while individuals with low social status are perceived as negative on the competence dimension and positive on the warmth dimension (Fiske et al., 2002; Kervyn et al., 2008). So far, the propositions of the SCM have received empirical evidence from multiple cultures, demonstrating good cross-cultural universality, including evidence from Confucian culture (Eckes, 2002; Cuddy et al., 2009; Asbrock, 2010; Binggeli et al., 2014; Yang et al., 2019). For instance, Chinese psychological scholars have found that participants not only evaluated HP individuals as cold on the warmth dimension in the explicit measure task, but also associated HP individuals with negative warmth traits in the implicit measure task (e.g., Implicit Association Task or Go/No-go Association Task), showing the same results pattern with the research conducted in Western culture (Fiske et al., 2002; Brambilla et al., 2011; Zhang et al., 2015; Yang et al., 2019).

Notably, while a large body of research demonstrates the cross-cultural universality of the SCM, some literature also reveals that the SCM shows cultural specificity in specific cultures (Stanciu et al., 2017; Grigoryev et al., 2019). For example, using the SCM as a theoretical framework, Stanciu et al. (2017) found that the contents of stereotypes about various of social groups in Romania varied with economic and social development existing in different regions, which was considered to indicate the within-cultural variations when the SCM was applied in Romania. Supporting the culture-specific proposition of the SCM, our research firstly uncovered the within-cultural variations of the SCM in Confucian culture. According to our findings, Chinese participants hold higher social responsibility for senior HP individuals than for junior HP individuals, and this difference of the expected social responsibility may cause participants to form more positive impression on the warmth dimension for senior HP individuals than for junior HP individuals. When combing our findings with the findings of prior research conducted in Confucian culture (Zhang et al., 2013, 2015; Wang and Yang, 2017; Yang et al., 2019; Li et al., 2022), we may can attempt to draw the conclusion that, on one hand, the SCM shows good cross-cultural universality of the SCM in Confucian culture; on the other hand, it may more or less display some Confucianism-specific characteristics. This conclusion, of course, is tentative and premature, but deserves our further investigation.

There were two things needing our further explanation. One thing was that, if people had different evaluation tendencies toward senior and junior HP individuals, why previous literature conducted in Chinese culture consistently revealed negative warmth perception toward HP individuals (Zhang et al., 2013, 2015; Wang and Yang, 2017; Yang et al., 2019). We attempted to explain this issue in terms of the accessibility theory (Förster and Liberman, 2007; Higgins, 2012). Specifically, prior literature concerning power stereotypes commonly classifies power into HP and LP individuals in a general way and does not intentionally differentiate senior and junior HP individuals. For example, Zhang et al. (2015) have applied the IAT to assess implicit power stereotypes about HP and LP individuals (Study 1) and found that participants implicitly associated HP individuals with negative warmth traits. In the study, they selected 8 HP labels and 8 LP labels as target words, and the selected 8 HP labels included: chairman, board chairman, boss, supervisor, leader, manager, director, and official. As for these HP labels, although chairman, board chairman, and boss can be easily classified into the senior HP subcategory, the others indeed are difficult to be clearly classified into the senior or junior HP subcategory. According to the propositions of accessible theory, those knowledge constructs with high accessibility tend to get priority in formation processing and guide individuals’ cognition and behaviors (Weick and Guinote, 2008; Higgins, 2012). We thus speculated that, in the case that no distinction between senior and junior HP individuals was explicitly mentioned, regardless for senior HP labels that participants knew little about them, or for those HP labels which were difficult to judge whether they belonged to the senior or junior HP subcategory, participants may extend the representation about junior HP individuals to the whole HP individuals, because they generally have more interacting experiences with junior HP individuals than senior HP individuals and the knowledge construct about junior HP individuals was thus more accessible (Li et al., 2022). This may be why past research conducted in Chinese culture consistently revealed negative warmth perception toward HP individuals.

Another thing deserving our further discussion was that on the intuitive level, the effects of power stereotypes on STI seemed to be smaller when behavioral actors were denoted as junior HP labels than when behavioral actors were denoted as senior HP labels across Study 3, 4 and 5 (Chinese sample). To some extent, this finding suggests that there may be more variations about stereotypes of junior HP individuals than stereotypes of senior HP individuals and this difference may be because people commonly had more contact experiences with junior HP individuals (Li et al., 2022). Past research concerning stereotypes demonstrates that contact experiences with the members of the stereotyped groups can effectively attenuate the stereotype effects in social cognition and judgments (Stangor et al., 1996; Ritter, 2013; Vezzali et al., 2015). As an example, with Italian elementary school children as participants, researchers conducted an intervention experiment whose aim was to examine whether intergroup contact experiences could modify participants’ original stereotypes toward immigrants (Vezzali et al., 2015). After a three-week intervention, the results showed that compared to the control condition (no intergroup contact training), intergroup contact experiences significantly reduced participants’ negative stereotypes toward immigrants. Given the above considerations, our explanation that more contact experiences with junior HP individuals lead to weaker stereotype effect in STI can be reasonable and acceptable.

### Limitations and future work

Several limitations existed in the current research. Firstly, by employing the theoretical framework of the SCM (Fiske et al., 2002), the present research defined competence and warmth as the two dimensions of stereotype contents. However, the warmth dimension actually can be subdivided into sociability and morality (Leach et al., 2007; Abele et al., 2021; Tanjitpiyanond et al., 2022). The former includes some trait reflecting one’s social relationships with others, such as likeable, warm, and friendly; the latter includes some traits reflecting one’s prosocial or cooperative tendency, such as honest, sincere, and trustworthy (Leach et al., 2007). According to this classification, the perception difference about senior and junior HP individuals intuitively is more likely to emerge on the sociability sub-dimension than on the morality sub-dimension. Indeed, in additional analyses with the data of Study 3 (specific results were provided in supplementary materials), by subdividing the warmth dimension into the sociability and morality sub-dimensions, we conducted a 2 (actor power: senior vs. junior) × 2 (warmth sub-dimension: sociability vs. morality) × 2 (trait valence: positive vs. negative) repeated-measures ANOVA. The results of data analyses have supported our speculation that target power induced the differences of STI mainly on the sociability sub-dimension rather than on the morality sub-dimension (specific data analyses were provided in supplementary materials). Given that a limited number of trials in Study 4 and 5 (12 experimental trials in total), we did not further explore the role of the sociability-morality sub-dimension, but we are conducting further works to systematically explore the role of the sociability-morality sub-dimension in the power stereotype effects in STI. Additionally, in future work, we can also attempt to explore the power stereotypes in STI on each independent trait rather than on the simple positive/negative dimension.

Secondly, our conclusion that senior HP individuals induced positive warmth perception and junior HP individuals induced negative warmth perceptions was drawn on the relative level but not on the absolute level. That means, the premise of positive warmth perception toward senior HP individuals was to choose junior HP individuals as the reference group, which would limit the generalizability of our findings to some extent. For example, Li et al. (2022) have compared participants’ warmth perception toward senior HP, junior HP, and LP individuals. The results showed that although participants indicated more positive warmth evaluations about senior HP individuals than junior HP individual, they actually indicated relatively negative warmth evaluations about senior HP individuals when the comparison occurred between senior HP and LP individuals. Given that, we may need to choose multiple reference groups for senior HP individuals and assess individuals’ warmth perception toward senior HP individuals more accurately.

Finally, when the false recognition is used to detect the occurrence of STI, a core premise of the paradigm is that the overall correct rate for recognition should exceed the chance level (higher than 50% in most cases; Todorov and Uleman, 2002). Given this consideration, following previous research (Wang et al., 2016), we predetermined that 24 trials (12 experimental trials and 12 filler trials) were included in the false recognition paradigm of Study 4 and 5. However, this limited number of experimental trials included in the false paradigm would more or less have an influence on the robustness and generalization of our findings. For example, the main effect of trait valence in the repeated-measures ANOVA, despite the fact that it was not our main focus, was significant in both Study 3 and 5, but was not significant in Study 4. We guess, this inconsistency might be because a small number of trials in each condition (3 trials) leaded to some unknown variations when performing data analyses. Given that, developing an ideal research paradigm in future research actually is necessary.

### Conclusion

Across five studies, we examined participants’ warmth perception difference about senior and junior HP individuals and the downstream effects on STI. The results showed that participants had more positive warmth perceptions for senior HP individuals than for junior HP individuals; as a result, participants were more likely to make STI from behaviors implying negative warmth traits when actors were junior HP individuals while participants were more likely to make STI from behaviors implying positive warmth traits when actors were senior HP individuals. As we have expected, perceived social responsibility accounted for the observed power stereotype effects in STI—the more social responsibility participants perceived about senior HP individuals, the stronger power stereotype effects they showed in STI. Notably, the different power stereotype effects in STI induced by senior and junior HP actors were observed only in Confucian culture, and in non-Confucian culture, participants tended to make STI from behaviors implying negative warmth traits regardless of senior and junior HP actors. The present research firstly demonstrated that the warmth perceptions about senior and junior produced different influences on STI in Confucian culture, and also enriched the understanding about the culture-specificity of the SCM.

## Data availability statement

The raw data supporting the conclusions of this article will be made available by the authors, without undue reservation.

## Ethics statement

The studies involving human participants were reviewed and approved by Taishan University. The patients/participants provided their written informed consent to participate in this study.

## Author contributions

FY and ML provided the research design. FY wrote and revised the manuscript. XF collected the data involved in the research and conducted data analyses. YH and QZ provided key suggestions for the revision of the manuscript. All authors contributed to the article and approved the submitted version.

## Funding

This research was supported by the Educational Science Project of Shandong Province (no. 2020QZD007).

## Conflict of interest

The authors declare that the research was conducted in the absence of any commercial or financial relationships that could be construed as a potential conflict of interest.

## Publisher’s note

All claims expressed in this article are solely those of the authors and do not necessarily represent those of their affiliated organizations, or those of the publisher, the editors and the reviewers. Any product that may be evaluated in this article, or claim that may be made by its manufacturer, is not guaranteed or endorsed by the publisher.

## Footnotes

^1^ www.credamo.com
